# Development of Gel-in-Oil Emulsions for Khellin Topical Delivery

**DOI:** 10.3390/pharmaceutics12050398

**Published:** 2020-04-26

**Authors:** Joana Pereira, Rita Gonçalves, Margarida Barreto, Clarisse Dias, Fátima Carvalho, António J. Almeida, Helena Margarida Ribeiro, Joana Marto

**Affiliations:** 1 Research Institute for Medicines (iMed.ULisboa), Faculty of Pharmacy, Universidade de Lisboa, 1649-003 Lisbon, Portugal; jir.pereira@campus.fct.unl.pt (J.P.); aalmeida@ff.ulisboa.pt (A.J.A.); 2 LEF Infosaúde, Rua das Ferrarias del Rei, 6, 2730 269 Barcarena, Portugal; rita.goncalves@anf.pt (R.G.); Margarida.Barreto@anf.pt (M.B.); Clarisse.Dias@anf.pt (C.D.); FatimaG.Carvalho@anf.pt (F.C.)

**Keywords:** gel-in-oil emulsions, cold process, hypopigmentation, khellin, topical drug delivery

## Abstract

Hypopigmentation is a progressive dermatological condition caused by a reduction in the skin pigment, melanin. Its treatment is considered a challenge due to the lack of a highly efficient single therapy. Currently, the main treatments include photochemotherapy, application of corticosteroids and immunosuppressants, and laser. Khellin-based gel-in-oil emulsions appear as a promising alternative since they ensure a concentration of the drug, a natural furanochromone, at the desired location, skin surface. Khellin promotes repigmentation as it forms a dark colored complex after solar irradiation. The aim of this study was the development and characterization (e.g., rheological behaviour, droplet size, tackiness, adhesion and spreadability) of three topical gel-in-oil emulsions prepared with different emollients, formulated through a cold emulsification process, and suitable for the incorporation of khellin. In vitro studies were performed to evaluate the drug release and permeation profiles across artificial membranes and excised human skin, respectively, using Franz-type vertical diffusion cells. The W/O emulsions developed showed macroscopic appearance, shear-thinning behavior with a mean droplet size from 3.28 to 4.28 μm, suitable for topical application. In vitro studies revealed permeation values of about 1% of khellin across the stratum corneum, making these gel-in-oil emulsions promising for preclinical and clinical studies. The cold process, being an easy and low energy production method, represents an innovative strategy to produce khellin-based gel-in-oil emulsions to treat patients with hypopigmentation.

## 1. Introduction

Vitiligo, a variant of hypopigmentation, is a progressive melanotic skin disorder that occurs through the decrease in melanin, the skin pigment. This dermatological disease affects 1%–2% of the world’s population, regardless of race, geographical location and gender. It is not a life-threatening condition, but it may cause severe disfigurement and considerable emotional and social distress [1,2]. This acquired disease is characterized by the sharply defined macules of de-pigmentation. Although commonly considered as a mere cosmetic problem, the implications of vitiligo on the quality of life of the patient are comparable with those of psoriasis on the emotional and socially functioning scale. The pathogenesis of these diseases is not yet fully understood, but it seems to be a multifactorial process involving hormonal factors, nutrient deficiency or genetic components, environmental risk factors, stress, infections derived from improperly administered skin treatments (e.g., laser peels, chemical peels, and others), pimples, blisters, chickenpox, scrapes and autoimmune linkages [3]. This makes the treatment of vitiligo a big challenge for dermatologists. Several therapeutic approaches in vitiligo are aimed at reversing the progressive loss of melanocytes and reconstituting the normal skin coloration. The most common treatment methods are topical corticosteroids, calcineurin inhibitors, systemic and topical psoralen ultraviolet-A light (PUVA), excimer laser, and broad- and narrow-band ultraviolet-B therapy (BB-UVB, NB-UVB). The management of vitiligo can also be done surgically. Another therapeutic approach to this disease is photochemotherapy combining the topical application of plant-derived flavonoids, such as khellin (KHE), with UVA (KUVA). The KUVA treatment leads to the formation of a low number of DNA cross-links, thus presenting low genotoxic potential [4].

KHE is a natural furanochromone derivative, the main active principle found in the seeds of *Ammi visnaga*, with a chemical structure closely resembling that of psoralen [3]. It is insoluble in water, soluble in ethanol, with a high octanol–water partition coefficient (Log *P* ≈ 3) [5]. KHE is used for the treatment of skin diseases characterized by the formation of multi-layered scales (psoriasis) or well-circumscribed achromatic macules (vitiligo) [6]. The therapeutic effects of KHE are directly linked to its photobiological activity, which in turn is related to its photobinding to DNA ability. When KHE is excited by UV radiation, it predominantly photobinds with thymidine in DNA, forming furan monoadducts, in contrast with psoralen compounds, which form diadducts and lead to interstrand crosslinks in the DNA duplex [7,8]. The process involves the intercalation of KHE between the base pairs of the nucleic acid followed by the photocyclo-addition with pyrimidine bases, particularly thymine [8,9,10]. The concentration of KHE required for the photoreaction with DNA is about 5–10 μg/mL [3,10].

Topical administration of KHE can offer the advantage of avoiding the adverse effects of oral administration, while also promoting drug accumulation at the correct skin site. KHE has been incorporated in topical formulations, mainly in liquid O/W emulsions [11,12]. Due their characteristics, such as low permeation, which keeps the substance active at the skin, liquid W/O emulsions play an important role in this type of pathology [13].

Concerning vitiligo, topical administration should ensure an active drug concentration at the target sites, i.e., undifferentiated melanocytes [3,11]. This route of administration is very attractive in the treatment of these diseases as it allows the application of the active substance directly to the skin at the desired location and at the required therapeutic concentrations [14,15]. Hypopigmented skin patches are not restricted to a particular part of human body, often showing symmetrical macules, usually increasing in number and size over time, and frequently appearing in visible areas like the face and extremities. Localized types of vitiligo include focal and mucosal vitiligo. The former refers to an isolated, small, depigmented patch (10–15 cm^2^) that lacks an obvious, unilateral segmental distribution. Thus, for the treatment with topical emulsions, by UV radiation, it is necessary to apply the formulation in localized areas regardless of size [16].

In addition, topical administration limits the need for systemically administered therapies and reduces adverse effects. Topical drug delivery systems feature a variety of dosage forms for cosmetic or dermatological application, including semi-solid (ointments, emulsions, gels) or liquid (lotions) [17,18], allowing a variety of therapeutic strategies that are crucial in complex disorders where individualized formulation may play a key role for success, whenever the patient’s specific needs are not met by the commercially available medicines. Hence, through the development of individualized compounding, the pharmacist plays an important role in deciding what is safe and appropriate for the patient’s needs, while always meeting regulated standards.

In this context, emulsions emerge as a promising approach for KHE delivery. These thermodynamically unstable systems consisting of two immiscible liquids, generally water and oil, have excellent solubilizing properties for lipophilic and hydrophilic active ingredients and great end-user acceptability due to the pleasant skin sensory [19]. Due to their characteristics, W/O emulsions play an important role in this type of pathology, since in the case of superficial skin lesions it is necessary to formulate an innovative vehicle that presents low permeation, keeping the substance active at the skin surface. Additionally, there is currently a preference for low energy development processes due to environmental issues. A gel-in-oil emulsion with high water content (above 80%) can be prepared using low shear cold emulsification, an environmentally friendly process [20,21]. A gel-emulsion is defined as an emulsion with a gel-like network structure and mechanical properties similar to a viscoelastic solid. A gel-emulsion is formed using an emulsifier and a polymer (gel network), which may occur due to the aggregation of the emulsion droplets or due to the gelation of the continuous phase [22]. Cold process emulsifiers are gaining more popularity as producers look for ways of decreasing costs and greener manufacturing methods. These emulsifying agents can be used at 4%–6%, allowing for the addition of different water- and oil-soluble actives. This method allows formulators to obtain extremely stable emulsions that feel light and silky on the skin and can be utilized with various oil phases including silicone and vegetable oils, and with polar solvents [23].

The aim of this study was the development and characterization (rheological behaviour, droplet size, tackiness, adhesion and spreadability) of three appropriate and innovative vehicles gel-in-oil emulsions to incorporate and deliver KHE on the skin surface. The incorporation of KHE through the cold process method was assessed and characterized, as well as the in vitro release and permeation studies. Thus, the development of a suitable and efficient W/O emulsion for topical application of 2% (*w*/*w*) khellin represents an innovative strategy to improve hypopigmentation therapy.

## 2. Materials and Methods

### 2.1. Materials

The khellin standard (4,9-dimethoxy-7-methyl-5H-furo[3,2-g][1]benzopyran-5-one) (≥98.0% by high-performance liquid chromatographic (HPLC)) was purchased from Sigma-Aldrich, St Louis, MO, USA. The raw material of khellin was purchased from Farmácia Alvide, Cascais, Portugal. SEPIPLUS™ 400 (polyacrylate-13 (and) polyisobutene (and) polysorbate 20 (and) sorbitan isostearate (and) water), EASYNOV™ (octyldodecanol (and) octyldodecyl xyloside (and) PEG-30 dipolyhydroxystearate), EMOSMART™ C28 (C21-28 Alkane) and EMOSMART™ V21 (C18-21 Alkane) were obtained from SEPPIC™ (La Garenne-Colombes, France). Isohexadecane was obtained from Massó (Barcelona, Spain). Purified water obtained by inverse osmosis (Millipore, Elix 3, Merck, Darmstadt, Germany) was filtered (pore 0.22 μm) and sterilized prior to use. Methanol and acetonitrile (both HPLC grade) were from Fisher. Tuffryn^®^ membranes (polysulfone membrane disc filters, 0.45 μm) were purchased from the Pall Corporation (East Hills, NY, USA).

### 2.2. Methods

#### 2.2.1. Emulsions Development

Three gel-in-oil emulsions (placebos), A, B and C, differing on the type of emollient (2.0%, *w*/*w*), were prepared according to Table 1. The emulsions were prepared by weighing and using a low shear cold emulsification process. Both phases were stirred until the emulsions completely homogenised. Then, an amount of 2.0% (*w*/*w*) of KHE was incorporated into the preformed emulsions, either manually using a mortar and pestle for 1 min or mechanically using an Unguator^®^ Pro equipment (Gako international, Germany) for 1 min and 5 min, at 25 °C.

#### 2.2.2. Physicochemical Characterization and Rheological Properties

The macroscopic appearance of each emulsion was visually analysed. A digital pH-Meter with a glass electrode (SevenEasy™ by Mettler Toledo, Columbus, OH, USA) was used to determine if the pH of the emulsions were within the specifications (pH 4.5–6.5).

##### Rotational and Dynamic Viscosity Measurements (Flow Curves)

Dynamic viscosity measurements were carried out between 1 and 1000 Pa on a logarithmic increment, ranging from 1.0 to 100 s^−1^, on 10 samples per decade. All measurements were performed at 25 °C, using cone and plate geometry (truncated angle 4° and radius 40 nm), 2 days after the preparation of the emulsions.

##### Oscillatory Dynamic Test

Structural experiments were performed with a controlled stress Kinexus Lab+ Rheometer (Malvern Instruments, Worcestershire, UK). Dynamic viscosity measurements were carried out between 1 and 1000 Pa on a logarithmic increment, ranging from 1.0 to 100 s^−1^, on 10 samples per decade. All measurements were performed at 25 °C, using cone and plate geometry (truncated angle 4° and radius 40 nm), 2 days after the preparation of the emulsions (*t* = 48 h). For the oscillation frequency sweep, the equipment was set to apply a frequency ranging between 0.01 and 100 Hz, on 5 samples per decade with a constant strain (0.1%), which was determined within the previously marked linear viscoelastic region of each sample.

##### Tackiness and Adhesion Test

To evaluate tackiness and adhesion properties, a pull away test was performed using plate and plate geometry. The parameters used were 0.1 mm/s for the gapping speed, 4 mm for the final gap and 0.2 mm for the working gap. The test was performed in three replicates of the same gel. All results were measured at 25 °C [24].

##### Spreadability

In this study, an adapted spreadability study was performed according to the procedure described elsewhere [25]. Briefly, 1 g of each emulsion was placed at the centre of a glass plate. This plate was covered with another glass plate of the same size, and a weight of 200 g was carefully applied on the upper face of the plate. After 1 min, the weight was removed and the diameter of the spread area (mm) was measured. The measurements were done in triplicate (*n* = 3), at room temperature.

#### 2.2.3. Droplet Size Analysis

The emulsions were analyzed for droplet size using an optical microscope equipped with an USB video camera (Bresser, Germany), at 25 °C. The droplet size was determined using an image analysis software (ImageJ^®^, 1.52v, NIH, Bethesda, MD, USA). Size data were expressed in terms of the relative size distribution of droplets and given as diameter values corresponding to percentiles of 10, 50 and 90. The span value was also taken into consideration. The span value is a statistical parameter useful for characterizing the wideness of the particle size distribution. The diameters of approximately 300 droplets for each emulsion were taken into consideration [26].

#### 2.2.4. In Vitro Release Studies of KHE-Containing Gel-in-Oil Emulsions

Release of KHE from the three candidate emulsions was measured using artificial membranes with a diffusion area of 1 cm^2^ under infinite dose conditions. The hydrophilic polysulfone membrane filters 0.45 µm (Tuffryn^®^) were mounted between the donor and receiver compartments on static vertical Franz-type diffusion cells. PBS was used as a receptor phase in perfect sink conditions throughout the experiment. The donor chamber contained 0.3 g of the respective emulsion. After the samples were applied on the surface of the membrane in the donor compartment, they were sealed with Parafilm^®^ to prevent water evaporation. The receptor phase was constantly stirred with a small magnetic bar and thermostated at 32 ± 0.5 °C throughout the experiments. Samples (0.2 mL) were collected from the receptor chamber at 1, 2, 3, 4, 5, 6, 7 and 8 h, placed into 1.5-mL vials for HPLC analysis, and replaced with the same volume of fresh buffer. Each set of experiments for each emulsion was performed in triplicate (*n* = 3) and the drug content was analysed by HPLC.

To study the release kinetics from emulsions loaded with KHE, Zero order (Equation (1)), First order (Equation (2)), Higuchi (Equation (3)) and Korsmeyer–Peppas (Equation (4)), models were fitted to the release data of the six emulsion [27]. The data obtained from in vitro release studies were computed using DDsolver, which is an Excel-plugin module, and different kinetic models were fitted to the resultant data, to obtain the best fit for the in vitro release:

*Q_t_* = *Q*_0_ + *K*_0_*t* (1)

where *Q_t_* is the amount of drug dissolved in the time t, *Q_0_* is the initial amount of drug in the solution and K_0_ is the zero-order release constant expressed in units of concentration/time.

(2) $$\log\;C=\log\;C_{0}-\frac{Kt}{2.303}$$

where *C_0_* is the initial concentration of drug, *k* is the first order rate constant, and t is the time. The data obtained were plotted as log (cumulative percentage of drug remaining) vs. time, yielding a straight line with a slope of $\frac{-K}{2.303}$.

(3) $$Q_{t}=\mathrm{KH}\;\times \frac{t1}{2}$$

where *Q_t_* is the amount of drug released in time t and KH is the Higuchi dissolution constant.

(4) $$\frac{Mt}{M\infty }=Kt^{n}$$

where $\frac{Mt}{M\infty }$ is a fraction of drug released at time *t*, *k* is the release rate constant and *n* is the diffusional exponent indicating the drug-release mechanism. The coefficient of determination (*R*^2^) was calculated for each model as it is an indicator of the model’s suitability for a given dataset.

#### 2.2.5. In Vitro Permeation Studies of KHE-Containing Gel-in-Oil Emulsions

Samples of adult human skin (24–43 years old) were obtained from breast reduction operations of Caucasian women and provided by an aesthetic surgery clinic. The skin was cut into discs and then sealed in aluminium foil and a plastic bag and stored at −20 °C. The skin discs were thawed in PBS pH 7.4 in a 60 °C bath for 1 min. Afterwards, the SC-epidermis layer of the skin was peeled off from the dermis using forceps [28]. The epidermis sheets were spread out in petri dishes filled with PBS for at least 30 min. The protocol complied with the Helsinki Declaration and Good Clinical Practice studies on topical products.

All skin sections were measured for transepidermal water loss (TEWL) at the beginning and end of the experiment, using a VapoMeter SWL5413 (Delfin Technologies, Kuopio, Finland).

The permeability of the drug through excised human skin was determined in vitro using a Franz diffusion cell system with a diffusion area of 1.0 cm^2^ in non-occluded conditions. The skin was mounted on the receiver chambers with the *stratum corneum* SC facing upwards, and the donor chambers were then clamped in place. The best undamaged skin fragments were carefully chosen. The receptor chamber contained 3 mL of PBS, pH 7.4, and the donor chamber contained about 0.4 g of each emulsion in order to completely cover the surface of the skin membranes. The receptor chamber content was stirred by a magnetic bar and the temperature of the cell was maintained at 32 ± 0.5 °C by a thermostatically controlled water bath, which was circulated through a jacket surrounding the cell body. Samples (0.2 mL) were removed from the receptor compartment for 28 h and an equal volume of fresh PBS solution was added. The collected samples were assayed for KHE by HPLC. The addition of PBS to the receiver compartment was performed with great care to avoid trapping air beneath the SC. A total of three different sources of skin were used, and for each one three replicates were tested.

All experiments were performed nine times (*n* = 9).

The total amount of KHE permeated through the excised human skin surface and into the receptor chamber was calculated and plotted as a function of time and determined by the following equation:

(5) $$Qt=\frac{Vr\times Ct+\sum_{t=0}^{t-1}Vs\times Ci}{S}$$

where *Ct* is the drug concentration of the solution contained in the receptor chamber at each sampling time; *Ci* is the drug concentration of the sample applied on the donor compartment; and *Vr* and vs. are the volumes of the receiver solution and the sample, respectively. *S* represents the skin surface area (1 cm^2^).

The drug steady-state flux, Jss, was calculated from the slope of the linear portion of the permeation curves and expressed as the mass of drug passing across 1 cm^2^ of skin over time. The permeability coefficients (*K_p_*, cm h^−1^) were obtained by calculating the quotient between the flux (J) and the initial drug concentration (*C_0_*) in the donor compartment applying the Fick’s 2nd law of diffusion (Equation (6)), and it was assumed that, under sink conditions, the drug concentration in the receiver compartment was not significant compared to that in the donor compartment.

(6) $$M\left(t\right)=klCC_{0}\left[\frac{Dt}{l^{2}}-\frac{1}{6}-\frac{2}{\pi^{2}}\sum_{n=1}^{\infty }\frac{{\left(-1\right)}^{n}}{n^{2}}\exp(-Dn^{2}\pi^{2}\frac{t}{l^{2}}\right]$$

#### 2.2.6. Quantification of KHE

The amounts of KHE incorporated in the formulations were determined using a high-performance liquid chromatographic (HPLC) method validated according to the ICH Q2B guideline [29], using a HPLC system (Pump and Autosampler Waters Separation Mode Alliance e2695 with Column Oven), provided with a Photodiode Array Detector Waters 2998 with a UV detector (UV–Vis spectrophotometric detector LC290, Perkin-Elmer) (Foster City, CA, USA), and a 250 × 4.6 mm, 5-μm reverse phase C18 column (Phenomenex Luna, , Torrance, CA, USA). The isocratic mobile phase consisted of water:acetonitrile (65:35 *v*/*v*) at flow-rate of 1.0 mL/min and the detection wavelength was set at 248 nm. The auto sampler chamber was maintained at room temperature and the run time was 20 min.

#### 2.2.7. Statistical Analysis

The statistical evaluation of data was performed using two or one-way analysis of variance (ANOVA) when appropriate, while Tukey–Kramer post-hoc multiple comparison tests were used to identify the significant differences between the groups. An α error of 5% was chosen to set the significance level, unless stated otherwise. Differences were considered significant when *p* < 0.05.

## 3. Results and Discussion

The main objective of this work was to develop KHE-containing gel-in-oil emulsions, able to remain and release their payload on the skin surface. Three gel-in-oil emulsions differing on the type of emollient (i.e., different chain length of the emollient) were prepared, all presenting a homogenous appearance, with bright white colour, with no phase separation phenomena.

Emulsions were prepared using a simple and flexible cold emulsification process, which saved energy and time. In this method, a ready-to-use thickening polymer (polyacrylate-13, polyisobutene, polysorbate 20) traps a high content of bulk water and a liquid emulsifier (octyldodecanol, octyldodecyl xyloside and PEG-30 dipolyhydroxystearate), stabilizing the water/oil interface. The oily phase contains different long chain alkanes as emollients and drug solubilizers. Long chain alkanes can inhibit coalescence, preventing interactions between emulsion droplets, thus increasing formulation stability. In addition, emulsion pH values were within the specifications (pH 4.5–6.5) being compatible with the skin.

### 3.1. Structural Analysis of Emulsions

**Figure 1:**
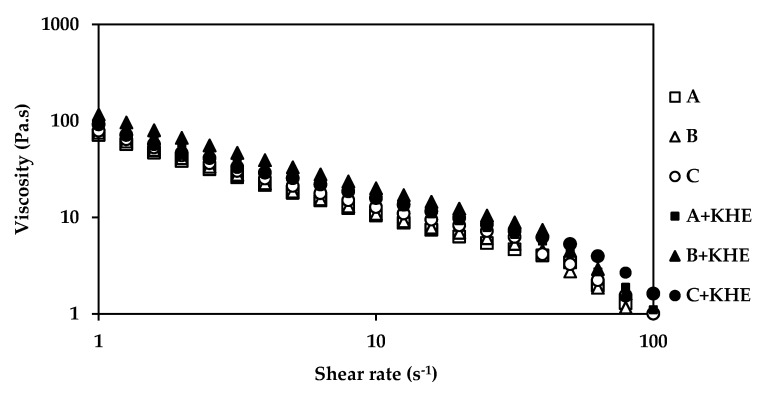
Effect of shear rate on apparent viscosity gel-in-oil emulsions A, B and C with khellin (KHE), and a comparison with the respective vehicles.

The rheological assessment of the gel-in-oil emulsions was performed by rotational and dynamic viscosity and oscillation frequency sweep tests. Continuous shear experiments (shear rate sweep, Figure 1) measure the ability of each system to resist structural breakdown during the standardized shearing procedure. All formulations present a non-Newtonian, shear thinning behavior, since the viscosity values decreased as the shear rate increased. The incorporation of the different emollients did not influence the structural elements of all formulations.

**Figure 2:**
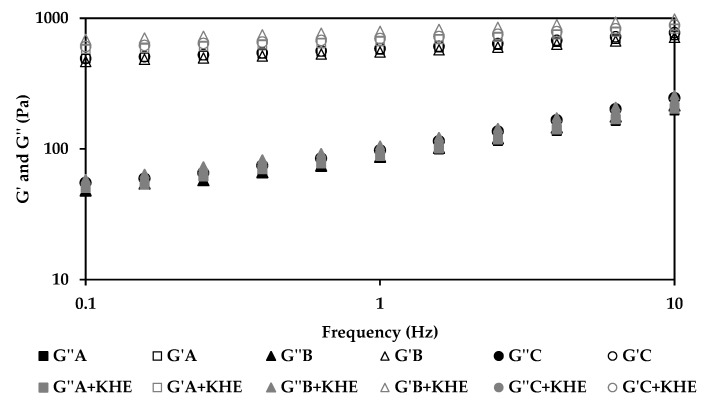
Effect of oscillation frequency sweep test of gel-in-oil emulsions A, B and C with KHE, and a comparison with the respective vehicles.

The rheological properties of all KHE-containing emulsions (Figure 1 and Figure 2) did not show significant differences compared to the placebo emulsions. The essence of viscoelastic analysis is to test various structures in the sample as long as the method does not destroy the structure. Varying the frequency of the applied waveform at low amplitudes will cause elements to respond when their characteristic times match the rate of change of stress associated with a particular frequency. Frequency test results are presented as G’ and G” moduli as a function of frequency (Figure 2). For all emulsions, the storage modules (G’) were higher than their loss modules (G’’), with a predominating “solid-like” behavior, revealing a prevalent viscoelasticity, that is in agreement with the viscosity measurements. All emulsions presented choesive structures indicating the presence of a strong polymeric microgel network structure probably due to polyacrylate (water phase polymer) and the hydration of PEG chains (from the emulsifier) [30,31,32]. The polymer present in the surfactant prevents water droplet sedimentation through the formation of a strong network [33,34]. The oscillation data show the same general trend for all formulations (Figure 2). At a specific frequency (1Hz), the oscillatory parameters for the formulation containing isohexadecane, are lower than those of C+KHE (containing C18-21 alkanes), indicating less structure, whereas the parameters for B+KHE (containing C21-28 alkanes) were in between. In fact, G’ and G’’ values, evaluated at 1 Hz, for A+KHE (containing isohexadecane, a C16 alkane), B+KHE (containing C21-28 alkanes) and C+KHE (containing C18-21 alkanes) were 557.2 and 85.7; 582.2 and 88.8; and 664.8 and 97.3, respectively. This is probably due to the emulsions’ droplet size, which in turn is influenced by emollient chain length.

Drug incorporation had no influence in the rheological behavior of the emulsions, as expected from a suitable drug delivery system.

The results from tackiness and spreadability are summarized in Table 2. The tackiness of a material is related to its stickiness and is due to cohesive forces in a material bridging two substrates, or the adhesive forces between two materials in contact. The adhesion assessment method aims to quantify the tackiness of structural film adhesives, by applying a force on the material, observing the time and the way the force is no longer exerted on it. This test allows for measuring the maximum force needed to break the resultant bond, the area under the curves that represents the adhesive/cohesive strength of the material, and the time required for the peak force to decay by 90% [35]. As expected, all emulsions presented similar results, emphasizing no influence of the emollient in these gel-in-oil emulsions. Although slight differences in strength were noticed, their force decay profiles were comparable, taking similar times for the force to decay by 90% of its peak value in the region of 2s. As a consequence, the emulsions presented high values of cohesiveness, justified by the polymer content.

### 3.2. Droplet Size Analysis

**Figure 3:**
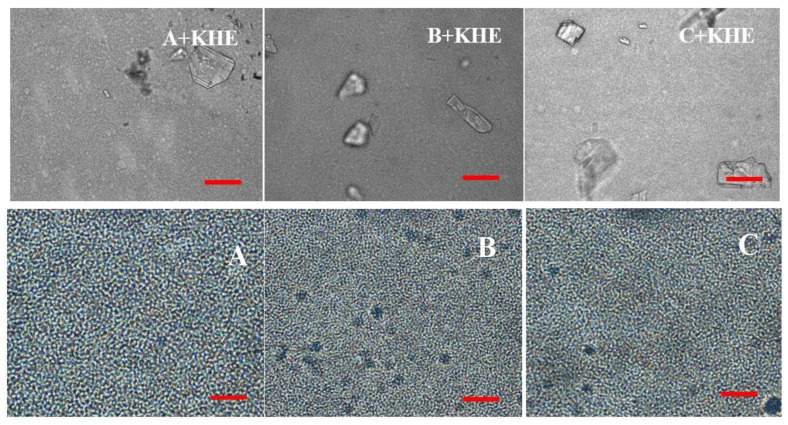
Micrographs of gel-in-oil emulsions A, B and C and of KHE-containing oil-in-gel emulsions (A+KHE, B+KHE, C+KHE) in bright light (Scale bar = 40 μm).

Representative micrographs of emulsions are shown in Figure 3. In bright light, all oil droplets are generally small, with emulsions B and C presenting slightly smaller droplets than emulsion A. Gel structures were also faintly prominent in these emulsions.

The drug icorporation induced a change in droplet size (Table 3), where KHE crystals are evident in all drug-containing emulsions (Figure 3; A+KHE, B+KHE, C+KHE). As observed, d(50) varied from 2.50 to 3.28, 2.19 to 4.28, and 2.17 to 3.42 for placebo emulsions and KHE-containing A, B and C emulsions. This increase is probably due to the composition of the oil phase of the emulsion. In fact, the droplet size increased with the length of the branched-chain aliphatic hydrocarbons: emulsion A+KHE (containing isohexadecane, a C16 alkane) presented a smaller droplet size than emulsions B+KHE (containing C21-28 alkanes) and C+KHE (containing C18-21 alkanes). The results also suggest that the chain length of the hydrocarbons slightly influenced the solid-state form of the drug, namely the size and number of crystals.

The data obtained from the droplet size agree with the micrographs, since emulsions containing KHE show a more distorted definition of the droplets with higher size, comparatively to placebos. The structure of emulsions regarding the shape of the droplets, the droplet size and polydispersity is of a great importance, affecting properties such as viscosity and stability [36].

### 3.3. KHE-Containing Emulsions: Homogenization and Dispersion Methods

**Figure 4:**
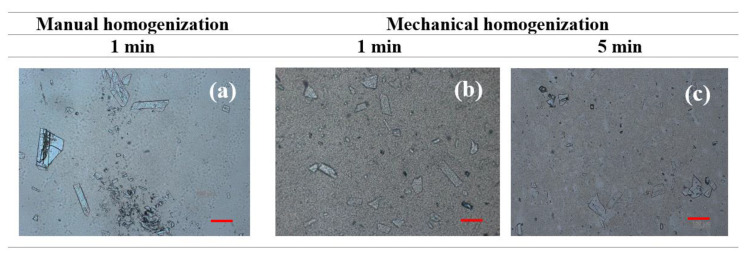
- Micrographs of oil-in-gel emulsion A after KHE incorporation for 1 min manually (**a**) and mechanically for 1 min (**b**), and for 5 min (**c**) at 25 °C (the scale bar corresponds to a length of 100 μm).

The efficiency of drug incorporation into the gel-in-oil emulsions was evaluated by comparing manual and mechanical agitation, using techniques currently used in pharmaceutical compounding. Manual force was exerted until the drug was completely incorporated, while, in the mechanical technique, a homogenizer (Unguator^®^ Pro) was used for periods of 1 and 5 min. Figure 4 shows the heterogeneity in size and distribution of KHE crystals in emulsions, indicating that homogenization time influences KHE particle size within the emulsion; after 5 min of incorporation, a decrease in crystal size was clearly noticed. Understanding and controlling the solid-state chemistry of active pharmaceutical ingredients is an important aspect of the drug development process because it allows one to adjust solubility, resulting in higher absorption, which is particularly relevant for water insoluble compounds, such as KHE.

### 3.4. In Vitro KHE Release

**Figure 5:**
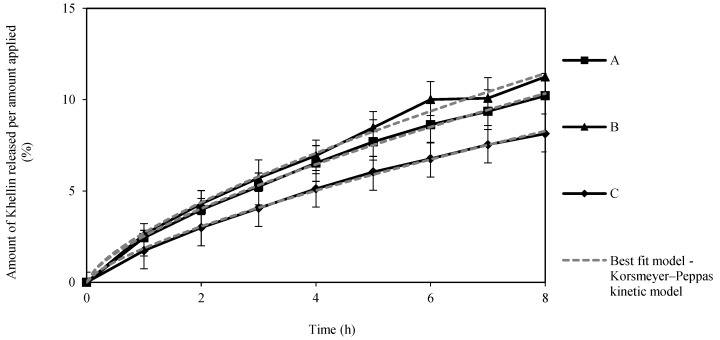
Release profiles of KHE from A, B and C oil-in-gel emulsions through Tuffryn^®^ membrane, during 8 h (mean ± SD, *n* = 3), to which the Korsmeyer–Peppas kinetic model was adjusted.

The in vitro release profiles shown in Figure 5 reveal a continuous KHE release up to 8 h, with no burst effect or plateau formation, suggesting a slow and sustained release for all three gel-in-oil emulsions. All three emulsions released about 10% of the drug after 8 h, with no significant differences in the amount of KHE released between the emulsions (*p* > 0.05).

The parameters obtained by fitting the models to the release profile data are listed in Table 4. The Korsmeyer–Peppas model showed the best fit for all emulsions, with the lowest Akaike Information Criterion (AIC) value. The release rate constant (*k*) suggests a faster drug release from gel-in-oil emulsion A [37,38,39]. Based on the diffusion exponent (*n*), the emulsions presented anomalous transport (non-fickian) mechanism for drug release [40]. These values suggest that the release was synergistically controlled by an anomalous diffusion mechanism of mass transport and polymer relaxation, such as matrix swelling, due to the presence of a polymeric microgel network (polyacrylate) [41,42,43]. Otherwise, in this particular study, the size of drug particles was not determinant since there is no plateau effect in release. Due to the faster drug release, gel-in-oil emulsion A was selected to continue for permeation studies.

### 3.5. In Vitro KHE Permeation

**Figure 6:**
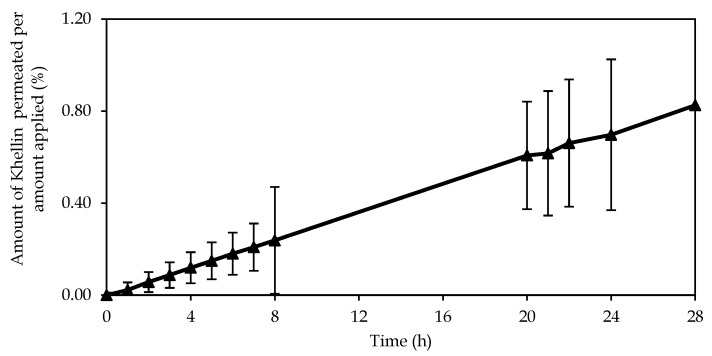
Permeation profile of KHE from gel-in-oil emulsion A through human *stratum corneum* (mean ± SD, *n* = 9).

The permeation results show that about 1% of KHE from emulsion A was able to penetrate the SC and viable epidermis (Figure 6). The permeation parameters were as follows: steady-state flux (Jss) 0.035 ± 0.010 μg/(cm^2^h), lag time 1.217 ± 0.34 h, and permeability coefficient (Kp) 1.33 × 10^−5^ ± 0.00 cm/h. As in the release studies, the cumulative amount permeated is in agreement with the rate of drug release. The emulsion presented a linear performance, which suggests that the determining factor is the amount of drug, independent of crystal size. As observed in the microscopical analyses of KHE-containing emulsions, the drug is dispersed, not completely dissolved, in the emulsion and therefore may not be as readily available to diffuse through the polymer matrix of the gel-in-oil emulsion. On the other hand, as KHE is not soluble in water and has a high Log *P* value, it has more affinity for hydrophobic areas, for lipid layers, and therefore for the external oil phase. On the other hand, the results obtained were as expected because the KHE gel-in-oil emulsion should stay at the skin surface, because a local action is required. To achieve this goal, the selection of the oily phase, in which different alkanes used as emollients, prevents drug cutaneous penetration and absorption. In addition, these emollients form a protective barrier on the skin surface, keeping KHE in the most superficial layers of the skin.

## 4. Conclusions

The results obtained in this work show that it was possible to develop a new topical vehicle for the treatment of hypopigmentation, in the form of innovative and attractive emulsions with a more pleasant and easy to spread texture, using a cold process easily performed by compounding in pharmacies and hospitals. The emulsions with polyacrylate and the hydration of PEG chains presented cohesive structures indicating the presence of a strong gel structure. Additionally, emollients with different carbon chain lengths had no influence on the structure of the emulsions. KHE-containing gel-in-oil emulsions presented macrostructures and a distribution of droplet size similar to that of the corresponding vehicles, suggesting that the developed emulsions were able to incorporate KHE without major structural changes. In terms of in vitro release and permeation, the developed emulsions showed similar profiles, with a drug release of about 10% after 8 h, and with a SC penetration of about 1% for KHE gel-in-oil emulsion A. Since the in vitro permeation studies were not performed with whole skin, the question of a possible complete skin permeation, although unlikely, still remains. Nevertheless, these studies will hopefully support the efficacy of these novel manipulated drugs for the administration of KHE to patients with hypopigmentation.

## Figures and Tables

**Table 1:** Quantitative and qualitative composition of gel-in-oil emulsions A, B and C.

Chemical Name	Function	Concentration (%, *w*/*w*)		
**Aqueous phase**	A	B		C
Water	Solvent	Qs100	Qs100	Qs100
Polyacrylate-13; polyisobutene; polysorbate 20; sorbitan isostearate; water ^1^	Emulsifier, Polymer	3.0	3.0	3.0
**Oil phase**				
Octyldodecanol; octyldodecyl xyloside, PEG-30 Dipolyhydroxystearate ^2^	Surfactant/ Emulsifier	3.0	3.0	3.0
Isohexadecane	Emollient	2.0	-	-
C21-28 alkane	Emollient	-	2.0	-
C18-21 alkane	Emollient	-	-	2.0

^1^ The breakdown of this ingredient is: polyacrylate-13 (50–70%); polyisobutene (22–32%); polysorbate 20 (2–7%); sorbitan isostearate (3–5%); water 0–10%)**;** ^2^ The breakdown of this excipient is: octyldodecanol: 55–65%, octyldodecyl Xyloside: 15–25% and PEG-30 dipolyhydroxystearate: 15–25%.

**Table 2:** Adhesive properties (mean ± SD, *n* = 6) and spreadability properties (mean ± SD, *n* = 3) of the gel-in-oil emulsions.

Emulsion	Peak Normal Force (N)	Time for Force to Reduce by 90% of Peak (s)	Area under Force Time Curve (N.s)	Diameter of Spread Area (mm)
A	−2.15 ± 0.034	−0.215 ± 0.0034	2.58 ± 0.12	48.3 ± 0.6
B	−1.95 ± 0.035	−0.195 ± 0.0035	2.27 ± 0.10	47.6 ± 0.4
C	−2.22 ± 0.039	−0.222 ± 0.0039	2.68 ± 0.14	46.7 ± 1.4

**Table 3:** Oil-in-gel emulsions’ droplet size distribution (mean ± SD; *n* = 3).

Emulsions	Droplet Size Distribution (μm)			
	d(0.1)	d(0.5)	d(0.9)	Span
A	2.00 ± 0.06	2.50 ± 0.07	3.08 ± 0.18	0.47 ± 0.20
B	1.70 ± 0.16	2.19 ± 0.17	2.76 ± 0.11	0.62 ± 0.14
C	1.66 ± 0.03	2.17 ± 0.06	2.79 ± 0.12	0.67 ± 0.13
A+KHE	2.51 ± 0.07	3.28 ± 0.08	4.33 ± 0.13	0.46 ± 0.15
B+KHE	2.48 ± 0.02	4.28 ± 0.03	5.76 ± 0.05	0.58 ± 0.09
C+KHE	2.38 ± 0.06	3.42 ± 0.08	5.16 ± 0.10	0.62 ± 0.15

**Table 4:** Kinetic parameters obtained by fitting the described models to the emulsions release data (mean ± SD, *n* = 3)**.**

Emulsion	Model	*K*	*R*^2^ Adjusted	AIC
**A**	**Zero-Order**	1.564 ± 0.15	0.884	18.171
	**First-Order**	0.017 ± 0.00	0.906	16.522
	**Higuchi**	3.734 ± 0.37	0.927	14.451
	**Korsmeyer–Peppas**	2.705 ± 0.29	0.98	4.630
		*n*: 0.69 ± 0.013		
**B**	**Zero-Order**	1.420 ± 0.19	0.883	16.089
	**First-Order**	0.015 ± 0.00	0.905	14.468
	**Higuchi**	3.395 ± 0.47	0.946	10.016
	**Korsmeyer–Peppas**	2.524 ± 0.45	0.997	−17.263
		*n*: 0.68 ± 0.032		
**C**	**Zero-order**	1.123 ± 0.16	0.914	9.425
	**First-Order**	0.012 ± 0.00	0.928	7.872
	**Higuchi**	2.674 ± 0.40	0.925	9.131
	**Korsmeyer–Peppas**	1.860 ± 0.53	0.997	−14.845
		*n*: 0.73 ± 0.08		

*K*—release constant; *R*^2^ adjusted—adjusted coefficient of determination; AIC—Akaike Information Criterion; *n*—release exponent.
